# PRMT5: splicing up tolerance

**DOI:** 10.1172/JCI185701

**Published:** 2024-10-15

**Authors:** Rathan Kumar, Parvathi Ranganathan

**Affiliations:** 1 Division of Hematology, Department of Internal Medicine, Comprehensive Cancer Center and; 2 Biological Sciences Graduate Program, The Ohio State University, Columbus, Ohio, USA.

## Abstract

Expression of tissue-restricted antigens (TRAs) within the thymus is critical for the establishment of self-tolerance; however, exact mechanisms regulating the expression of TRAs has proven more complex than previously appreciated. In this issue of the *JCI*, Muro et al. identify a central role for protein arginine methyltransferase 5 (PRMT5) in posttranscriptional regulation of TRAs and thereby central tolerance. Using conditional KO mice, the authors showed that thymic deficiency of *Prmt5* predisposed mice to developing autoimmune diseases while enhancing antitumor efficacy. These studies provide insight into the role of PRMT5 in shaping the T cell repertoire with implications for the development of therapies.

## Protein methyltransferase 5

Posttranslational modifications via arginine (R) methylation are catalyzed by a group of enzymes called protein arginine methyltransferases (PRMTs) and play an important role in gene transcription, cell-cycle progression, signal transduction, and mRNA processing (1–3). PRMT5, a type II PRMT, symmetrically dimethylates arginine residues on histones and nonhistone proteins.

PRMT5 is unique among PRMT enzymes in that the cofactor, methylosome protein 50 (MEP50), increases PRMT5’s methyltransferase capabilities by increasing substrate affinity (1, 4). Symmetrical dimethylation of the Sm proteins D1, D3, and B/B′ on their C-terminus by the PRMT5-MEP50 complex regulates splicing of nuclear RNA into messenger RNA (mRNA), thereby regulating RNA processing (3). PRMT5 has been investigated for its role in hematopoiesis, lymphocyte function, and oncogenesis (5). Within the T cell compartment, PRMT5 is required for the maintenance, survival, and proliferation of conventional CD4^+^ and CD8^+^ T cells as well as invariant NK T cells (6, 7). Loss of PRMT5 affects Th17 differentiation via the cholesterol synthesis pathway (8). Using genetic KO and pharmacological inhibitors of PRMT5, several studies have also shown PRMT5 as a viable target for autoimmune disorders (8, 9) and alloimmune disorders such as graft-versus-host disease (GVHD) (10). However, while PRMT5 is important for T cell function, whether PRMT5 has a role in establishing tolerance had not yet been investigated. In this issue of the *JCI*, Muro et al. (11) tackle this question by investigating the role of PRMT5 during thymic selection.

## Thymic selection and establishment of central tolerance

The thymus is an essential organ that supports the development of a diverse and self-tolerant T cell repertoire. Expression of the surface coreceptor molecules CD4 and CD8 distinguishes the progression of thymocyte development from double-negative (DN) to double-positive (DP), and finally single-positive (SP) T cells. As the thymocytes undergo maturation, they traffic through the thymus, from the outer cortex to the inner medulla (12). Thymic epithelial cells (TECs) mediate central tolerance, with cortical TECs (cTECs) presenting ubiquitous self-antigens facilitating positive and negative selection of DP thymocytes, which then traffic to medullary TECs (mTECs). mTECs are key in displaying a diverse array of tissue-restricted antigens (TRAs) that (a) mediate negative selection and deletion of autoreactive SP thymocytes, (b) successfully facilitate the development of natural regulatory T cells (nTregs), and (c) influence the maturation of functional naive SP CD4^+^ and CD8^+^ T cells (13). The output of a mature, self-tolerant, and diverse T cell repertoire is dependent on mTEC expression of TRAs (14).

An important subset of mTECs mediate TRA presentation via the transcription factor autoimmune regulator (AIRE) (15). AIRE deficiency was initially discovered in autoimmune polyglandular syndrome type 1 (APS1), a multiorgan autoimmune disorder, (16, 17), and studies using *Aire*-KO mouse models recapitulated this finding (14, 16, 18). In APS1, patients develop a range of organ-specific autoimmune diseases throughout their lifetime, starting in early childhood (17). Furthermore, loss of AIRE^+^ mTECs during acute GVHD leads to impaired negative selection of self-reactive T cells that mediate chronic GVHD (19). In addition to temporal regulation of AIRE, posttranscriptional mechanisms also dictate AIRE expression (18, 20). Thus, delineating AIRE-dependent and -independent TRA expression is fundamental to understanding the mechanisms of T cell selection. TNF family members RANK and RANK-L are required for medullary thymic development (21). Recent studies using RANK-L blockade have shown that inhibition of RANK signaling limits AIRE and tissue-specific antigen (TSA) expression within mTECs and rescues tumor-specific T cells from negative selection, resulting in decreased tumor burden (22). Furthermore, *Aire^–/–^* mice have synergistic tumor control with several immune checkpoint blockades (23). Thus, loss of central tolerance through *Aire* deficiency improves antitumor function via retention of self-recognizing T cell receptor–bearing (TCR-bearing) T cells.

Muro and authors (11) investigated how PRMT5 regulates TRA expression through AIRE-dependent and -independent mechanisms. Modulation of PRMT5 expression can influence the diversity of TCRs that could hold therapeutic potential for cancers and autoimmune disorders.

## PRMT5 regulates TRA expression during thymic selection

Using a TEC *Prmt5* conditional-KO (*Prmt5*-cKO) mouse model (*Foxn1*-CRE-*Prmt5^fl/fl^*), Muro and colleagues (11) present compelling evidence that mTEC-specific PRMT5 expression is important for TRA expression. The investigators found smaller thymuses, reduced CD80 and MHC class II expression, and lower absolute counts of the mature mTEC population in *Prmt5*-cKO mice compared with WT mice. Despite diminished antigen presentation, there were no differences in DN, DP, or CD4 SP thymocyte frequencies, with only a minimal decrease in CD8 SP and Treg compartments. Next, researchers investigated Aire expression in *Prmt5-*cKO mTECs, observing deficient mRNA processing via increased intron 10 retention in misspliced *Aire* mRNA correlating to a marked decrease in AIRE protein expression. This finding suggests that PRMT5 regulates AIRE expression and thus antigen presentation in mTECs.

Given this relationship between the two genomic regulators — PRMT5 and AIRE, Muro et al. (11) aimed to further delineate mechanisms driving TRA expression in mTECs. Using bulk RNA-Seq of functionally mature mTECs (mTEC^hi^), the investigators discovered highly similar TRA gene expression patterns between *Prmt5*-cKO and *Aire*-KO mTECs, each signature grouped individually as TRA^PRMT5^ and TRA^AIRE^, respectively. Both groups shared downregulation of 651 TRA predicted genes (termed TRA^Shared^). TRA^AIRE^ and TRA^PRMT5^ had a sharp increase in intron retention and thus diminished mRNA processing and antigen presentation capabilities. Interestingly however, *Aire*-KO mTECs had increased PRMT5 expression and symmetrical dimethylated arginine residues, indicating functional PRMT5 with decreased TRA expression. Thus, mTEC expression of TRA is dependent on functional mRNA splicing, which PRMT5 and AIRE regulate by both overlapping and unique mechanisms.

## Loss of thymic PRMT5 in disease

**Figure 1:**
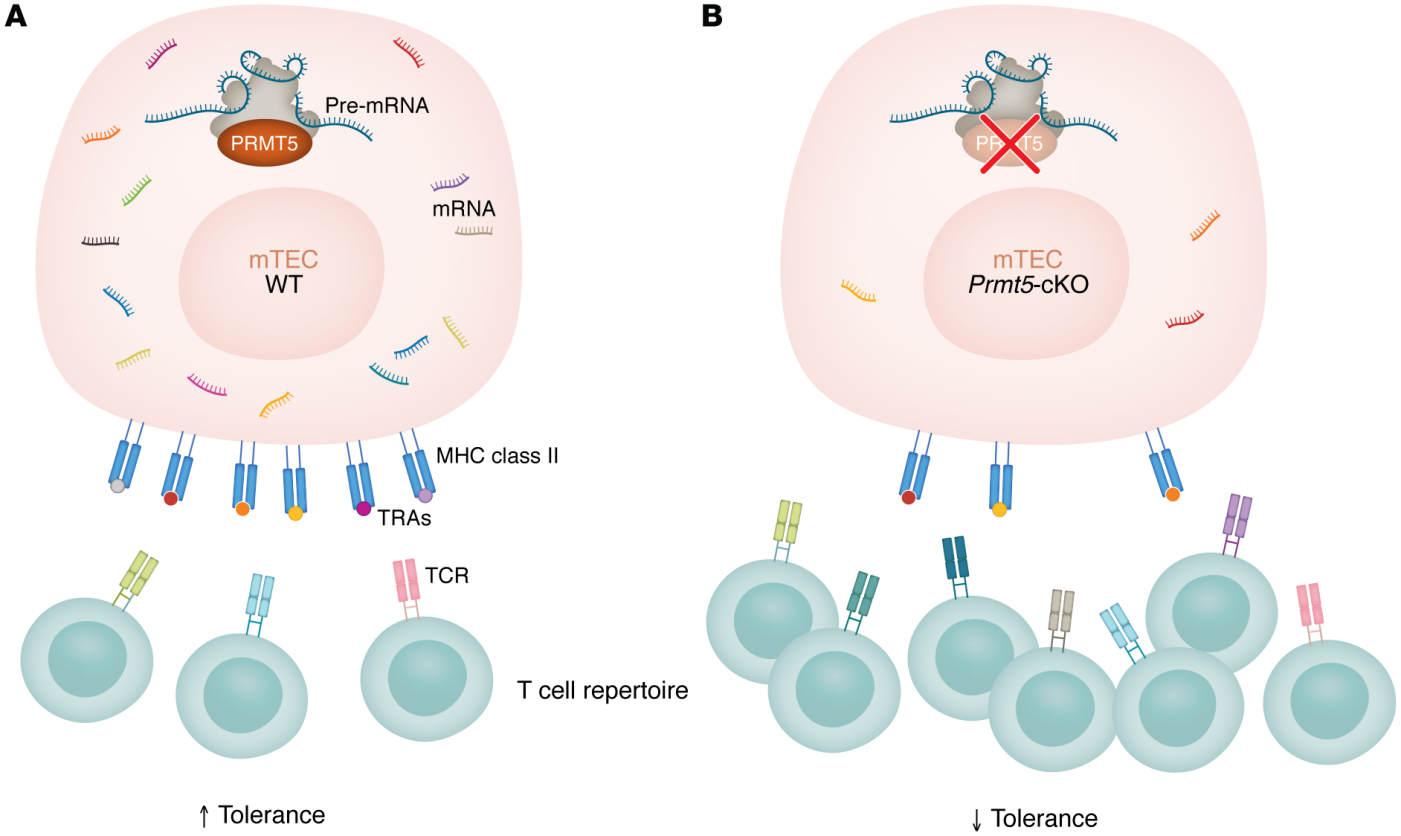
Muro et al. present a role of mTEC-expressed PRMT5 in shaping immune tolerance. (**A**) In mTECs expressing WT PRMT5, intact splicing generates a diverse range of TRA expression and presentation, resulting in clonal deletion of self-reactive T cells and the generation of a self-tolerant T cell repertoire. (**B**) Conditional deletion of *Prmt5* in mTECs results in reduced spliceosome efficiency and increased intron retention, leading to decreased TRA expression. The resulting T cell repertoire included self-reactive T cells and thus a predisposition toward autoimmunity as well as enhanced antitumor function.

Muro and authors next sought to determine the pathologic relevance of diminished TRA expression in *Prmt5*-cKO mice. Using deep sequencing of TCRs from sorted mature CD4 SP and CD8 SP cells, Muro and colleagues (11) discovered that *Prmt5*-cKO mice contained a different TCR repertoire compared with healthy control mice (Figure 1). Furthermore, serum from the *Prmt5*-cKO mice contained antibodies against multiple organs including the kidney, liver, lung, salivary gland, and pancreas — classical target organs in various autoimmune disorders. These findings show that thymic epithelial cell *Prmt5* deficiency mediates loss of T cell tolerance and promotes the generation of autoreactive B cells and antibody production.

Furthermore, Muro and authors noticed that lungs of *Prmt5*-cKO mice had a large infiltration of mature CD4^+^ and CD8^+^ T cells. With the previous discovery of diminished TRA expression, a diverse TCR repertoire, and increased lymphocytic lung infiltration, the authors hypothesized that *Prmt*5-cKO T cells may display improved antitumor efficiency due to retention of tumor antigen–reactive T cells. To examine this possibility, Muro et al. (11) revisited the TRA sequencing data, observing decreased expression of the common melanoma antigens gp100, tyrosinase, TRP-1, and TRP-2 in *Prmt5*-cKO mTECs compared with the control. The researchers tested antitumor immunity by intravenously transplanting B16F10 melanoma cells and measuring lung metastasis over two weeks via bioluminescence. *Prmt5*-cKO mice had decreased tumor growth accompanied by increased infiltration of IFN-γ–producing CD8^+^ T cells in the lungs, suggestive of superior tumor control.

## Implications and future directions

The findings presented in the Muro et al. study (11) provide mechanistic insight into the regulation of TRA expression in mTECs during negative selection and establishment of a self-tolerant T cell repertoire. Tissue-specific loss of PRMT5 in TECs resulted in loss of thymic structure, decreased AIRE expression, and decreased antigen presentation. *Prmt*5-deficient mTECs had diminished mature mRNA accompanied by an increase of unspliced pre-mRNA, ultimately resulting in a decrease of the diversity of antigens expressed. There was downregulation of TRA^PRMT5^ within the mTEC^hi^ compartment resulting in the development of an autoreactive TCR repertoire. While these nontolerant T cells had enhanced antitumor immunity, they also promoted the production of autoreactive antibodies against multiple antigens and organs, highlighting the double-edged nature of shaping the TCR repertoire.

The results of Muro et al. (11) give rise to important mechanistic and therapeutic implications. First, the authors identified regulation of mRNA processing and TRA expression via PRMT5-mediated mRNA splicing. However, given the wide substrate repertoire of PRMT5 including histones, it is likely that PRMT5 modulates TRA expression through multiple pathways in addition to splicing. Further investigation into the function of PRMT5 within mTECs would help expand our understanding of the establishment of central tolerance. The loss of tumor antigen expression in mTECs of *Prmt*-cKO mice resulted in enhanced tumor control and decreased metastasis. These results support the finding by Muro et al. that cell-type–specific PRMT5 inhibition has therapeutic implications in cancer treatment, although additional tumor models need to be tested for preclinical efficacy. Furthermore, there has been a growing interest in pursuing PRMT5 inhibition as a therapeutic agent for inflammatory diseases such as autoimmune disorders and GVHD (5, 8–10, 24). While PRMT5 inhibition decreases cytokine production and proliferation of autoreactive T cells (9), it simultaneously allows for maturation of these T cells. Thus, the work by Muro et al. highlights the importance of identifying modes of tissue-specific PRMT5 inhibition that will allow for increased translational therapeutic relevance.

## Version 1 10/15/2024

Electronic publication
